# Self-reported Deposits Versus Actual Deposits in Online Gambling: An Empirical Study

**DOI:** 10.1007/s10899-023-10230-1

**Published:** 2023-07-04

**Authors:** Michael Auer, Niklas Hopfgartner, Denis Helic, Mark D. Griffiths

**Affiliations:** 1 neccton GmbH, Davidgasse 5, Müllendorf, 7052 Austria; 2 Institute of Interactive Systems and Data Science, Graz University of Technology, Sandgasse 36/III, Graz, 8010 Austria; 3 School of Applied Data Science, Modul University Vienna, Am Kahlenberg 1, Vienna, 1190 Austria; 4 International Gaming Research Unit, Psychology Department, Nottingham Trent University, 50 Shakespeare Street, Nottingham, NG1 4FQ UK

**Keywords:** Gambling, Monetary depositing, Responsible gambling tools, Problem gambling, Self-assessment, Personalized feedback

## Abstract

In recent years a number of studies have used objective gambling data from online gambling operators to study gambling behavior. A few of these studies have compared gamblers’ actual gambling behavior (using account-based tracking data) with their subjective gambling behavior (using responses from survey data). The present study extended previous studies by comparing self-reported money deposited with the actual amount of money deposited. The authors were given access to an anonymized secondary dataset of 1,516 online gamblers from a European online gambling operator. After removing those who had not deposited any money in the previous 30 days, the final sample size for analysis was 639 online gamblers. The results indicated that gamblers were able to estimate fairly accurately how much money they had deposited in the past 30 days. However, the higher the amount of money deposited, the more likely gamblers underestimated the actual amount of money deposited. With respect to age and gender, there were no significant differences between male and female gamblers in their estimation biases. However, a significant age difference was found between those who overestimated and underestimated their deposits, with younger gamblers tending to overestimate their deposits. Providing feedback as to whether the gamblers overestimated or underestimated their deposits did not lead to any additional significant changes in the amount of money deposited when considering the overall reduction in deposits after self-assessment. The implications of the findings are discussed.

## Introduction

The majority of gambling studies rely on self-report data, such as studies used to estimate the prevalence of gambling participation (e.g., King et al., 2020; Gómez et al., 2020) and prevalence of problem gambling (e.g., Chóliz et al., 2021; Hing et al., 2022; Williams et al., 2021). Hodgins and Makarchuk (2003) tested the reliability and validity of self-reported gambling behavior among two samples of problem gamblers. They found that there was a high agreement for self-reported number of days gambling and money spent gambling over a six-month timeframe between a two- to three-week retest period. They also found that gamblers reported significantly more gambling at the second interview.

Currie et al. (2006) computed low-risk gambling thresholds based on self-reported gambling intensity and problem gambling among 19,012 Canadians. They concluded that gamblers should not play more than two to three times per month, spend less than $501–1000 CAN per year on gambling, and spend less than 1% of gross family income on gambling activities. Players who did not exceed these thresholds were less likely to be problem gamblers. A subsequent longitudinal study to identify low-risk gambling thresholds by Currie et al. (2017) based on self-report data from 3,863 Canadian adults suggested a maximum gambling frequency of eight times per month, $75 CAN total per month, and 1.7% of income spent on gambling were low-risk gambling thresholds. All derived thresholds were higher than the previously derived limits from cross-sectional data. Gamblers who exceeded any of the three low-risk limits were four times more likely to experience future harm than those who did not.

### Player Tracking Data in Gambling Research

Several studies have used player tracking data to gain a better understanding of online gambling behavior (e.g., Ukhov et al., 2021; Luquiens et al., 2016; Dragicevic et al., 2015; Percy et al., 2016). In a review of player tracking research in online gambling, Chagas et al. (2017) concluded that empirical analysis of gambling behavior using tracking data had contributed significantly to the understanding of player behavior. For example, studies have investigated voluntary self-exclusions in online gambling (e.g., Dragicevic et al., 2015; Percy et al., 2016; Hopfgartner et al., 2023a, b) and found that voluntary self-exclusion can quite accurately be predicted based on player tracking data. Hopfgartner et al. (2023b) found that online self-excluders who returned to gamble, continued to spend as much money as they did before the self-exclusion, and concluded that self-excluders who return to gamble should be monitored by gambling operators.

Additionally, a number of studies have correlated player tracking data with responses to problem gambling screens (Murch et al., 2023; Auer & Griffiths, 2022; Luquiens et al., 2016; Louderback et al., 2021). These have reported significant correlations between self-reported problem gambling and player tracking data.

### Self-reported Versus Actual Gambling Behavior

In recent years, a number of studies have used objective gambling data from online gambling operators to study gambling behavior (e.g., Auer and Griffiths, 2023; Ghaharian et al., 2023; Perrot et al., 2022). However, only few studies have explored the accuracy of self-reported gambling intensity by comparing it with objective gambling data of real-world online gamblers (i.e., Auer & Griffiths, 2017; Braverman et al., 2014; Heirene et al., 2022). Braverman et al. (2014) analyzed actual betting data and self-reported gambling expenditure, as well as self-reported problem gambling from 2,259 online gamblers. Participants were asked how much money they had lost during the past three and 12 months on fixed odds betting, casino gambling, and live betting. Gamblers estimated their three-month losses more accurately than their 12-month losses, and gamblers with gambling-related problems showed more discrepancies between self-reported and actual losses. Across games, half of the gamblers overestimated their losses, and 23%–48% of the gamblers underestimated their losses. Braverman et al. (2014) defined a bias score as being the self-reported net outcome minus the actual net outcome divided by the average bet size. A bias score of within − 1 and + 1 was determined as an accurate recall. Only 13% and 7% of gamblers accurately recalled their past three-month and 12-month outcomes, respectively.

Auer and Griffiths (2017) had access to self-report data for one-month losses or wins from 1,335 Norwegian online gamblers as well as the actual money lost by the same players for the same time period. Overall, the median amount lost, and the estimated amount lost were quite similar. However, the estimation bias increased with the intensity of play and the types of games played. The findings suggested that players with higher losses had more difficulty estimating their gambling expenditure. The same study also found that gamblers who engaged in high event frequency games (e.g., casino-type games) were less accurate in estimating their losses compared to players who engaged in low event frequency games (e.g., lottery games). Overall, large self-reported losses also indicated large actual losses. Auer and Griffiths (2017) used the same aforementioned bias score as Braverman et al. (2014) and found that 74% of gamblers correctly estimated their one-month loss. The larger percentage of players accurately assessing their loss was attributed to the shorter time period than in Braverman et al.’s study.

Heirene et al. (2022) compared self-reported past 30-day net outcome and the number of bets with actual gambling data among 514 Australian sports bettors who reported their net outcome. Participants had to report either the amount won or lost. They found that only 21 bettors were accurate within a 10% margin of their actual outcome (4.09%). Only two bettors were completely accurate (0.39%). Two-thirds of bettors underestimated their losses (n = 333; 64.79%). Lower actual net losses were associated with greater under- and over-estimation of losses. Out of 652 participants who reported their gambling frequency, 48 were accurate within a 10% margin of their actual frequency (7.36%). Most participants (n = 454) underestimated their number of bets (69.63%).

### Reliability Challenges in Self-assessments

Heirene et al. (2022) argued that one potential explanation for the bias between self-reported money spent and actual money spent could be the complexity of calculating the loss or win. For example, a gambler could place a bet of €50 on a roulette number and win €1,750. If the gambler then placed €1,800 on another roulette number and lost, the total loss of the two games would be €50. Similarly, after winning €1,750, the player could have wagered only €1 per game and therefore played much longer until reaching an account balance of zero. In this case, it would be even harder for the player to calculate the total loss across hundreds of games played. Furthermore, gamblers can deposit and withdraw money into their gambling account at any time. Consequently, when players are asked how much money they have lost or won in a month or a longer period of time, it can be understandably difficult to give an accurate estimate. Blaszczynski and Dumlao (1997) tested how participants calculated money spent based on an example which was provided. The example presented to participants was: *“You recently decided to gamble $120 on your favourite form of gambling. You initially won $60 but then following a bad run of luck, lost $100. Feeling tired, you decided to leave and return home”.* Participants calculated money spent differently with 64% responding $40 which is the net expense strategy [i.e., $120-($120+$60-$100)], 17% responding $120 which is the stake, and a smaller number of participants responding $160 (which is equal to $120+$100-$60). The study by Blaszczynski et al. (1997) showed that players can interpret questions about money spent differently in absence of specific instructions.

Self-assessed spending might also be impacted by a recall bias. Gilovich (1983) found that sports bettors spent more time explaining away their losses than their wins. They tended to discount their losses but “bolstered” their wins. This might be especially applicable to the loss metric because single large wins can distort a gambler’s assessment of the net outcome over a period of time. Another reason for a potential inaccuracy in self-assessments lies in each gambler’s motivation to report accurate estimates. In an online study of self-reported problem gambling, Auer and Griffiths (2022) measured the time taken to answer questions on the Problem Gambling Severity Index (Ferris & Wynne, 2001) in a sample of 1,287 online gamblers. They found that gamblers who took very little time to answer the questions frequently chose the most extreme answer categories. This indicates that some gamblers do not pay attention to the questions and that their answers are therefore not reliable.

Recent research has also shown that personalized feedback can lead to reduced monetary spending (e.g., Auer and Griffiths, 2015, 2018, 2020; Wohl et al., 2017). Wohl et al. (2017) asked 649 land-based gamblers how much they thought they had won or lost over a three-month period while using their loyalty card. They were then told how much they had actually lost. After three months, gamblers were again asked how much they thought they had lost in the previous three months. Gamblers who had underestimated their losses did not think that their amount of money spent had reduced. However, in reality, gamblers who had underestimated their losses spent less money in the three months after. Wohl et al. (2017) concluded that the personalized feedback about the actual money lost had an impact on subsequent money spent. In a study by Auer and Griffiths (2018), 11,829 players Norwegian online gamblers had access to their personal gambling expenditure and were asked whether they thought the amount they lost was (i) more than expected, (ii) about as much as expected, or (iii) less than expected. It was hypothesized that gamblers who thought that the actual amount of money spent was more than expected would subsequently spend less money. This was found to be true in specific subgroups of gamblers. Auer and Griffiths (2015) analyzed the impact of personalized inbox messages and information about money spent on subsequent money wagered and time spent among 15,216 Swedish online gamblers. The results showed that online gamblers receiving personalized feedback spent significantly less time and money gambling compared to controls that did not receive personalized feedback. The same finding was also replicated in another later study (i.e., Auer and Griffiths, 2020).

### The Present Study

The present study aimed to replicate previous studies by comparing self-reported money spent with the actual money spent. Due to the aforementioned difficulties in estimating the amount of money lost, the present study compared the self-reported amount of money deposited with the actual amount of money deposited. The amount deposited refers the amount of money that players transferred into the online gambling account. Various payment methods such as bank transfer, credit card, and many others can be used. Typically, there are far fewer depositing events than wagers, which arguably helps gamblers keep better track of the total amount of deposits. Moreover, the amount of money deposited aligns with the amount of money lost over time. Therefore, it was hypothesized that gamblers’ estimates of their monetary deposits would be more accurate than compared to previous studies. Moreover, the present study aimed to answer the following research questions (RQs):

**RQ1** Can gamblers accurately estimate their total amount of money deposited in the past 30 days?

**RQ2** Are age and gender associated with the accuracy of monetary deposit amount estimation?

**RQ3** Does the gambling behavior impact the accuracy of monetary deposit amount estimation?

**RQ4** Does feedback about actual money deposited impact subsequent monetary depositing?

## Methods

The authors were given access to an anonymized secondary dataset of 1,516 online gamblers from a European online gambling operator. At the time of the study, the gambling operator’s product portfolio consisted of online casino games such as slots, roulette, and blackjack. To estimate amount the amount of money deposited over the past 30 days, players had to navigate to a specific section of the online gambling site. After they had estimated the amount of money they had deposited, they were then informed about the actual amount of money they had deposited.

**Fig. 1:**
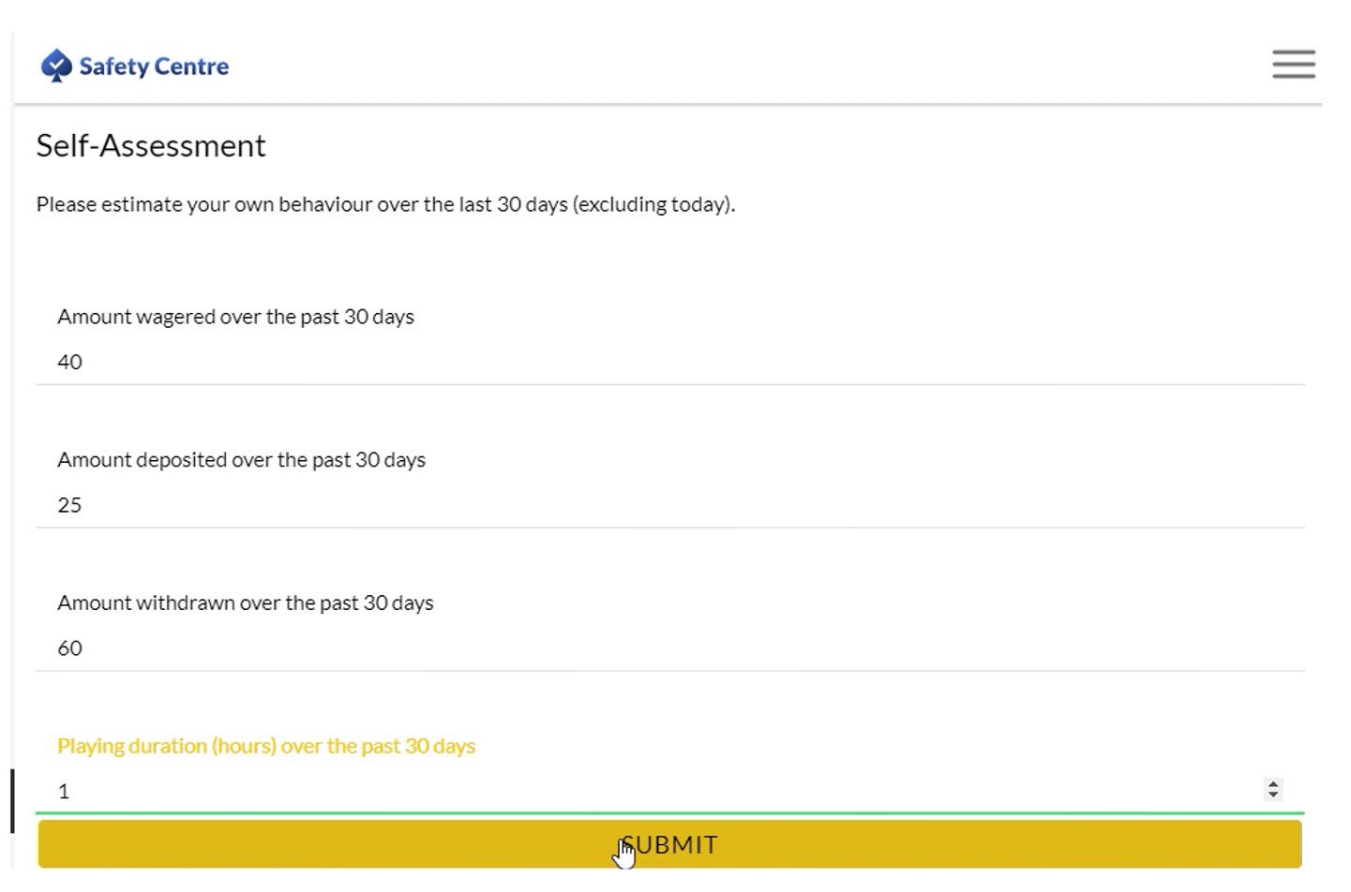
Self-assessment of the amount of money deposited on the online gambling website

**Fig. 2:**
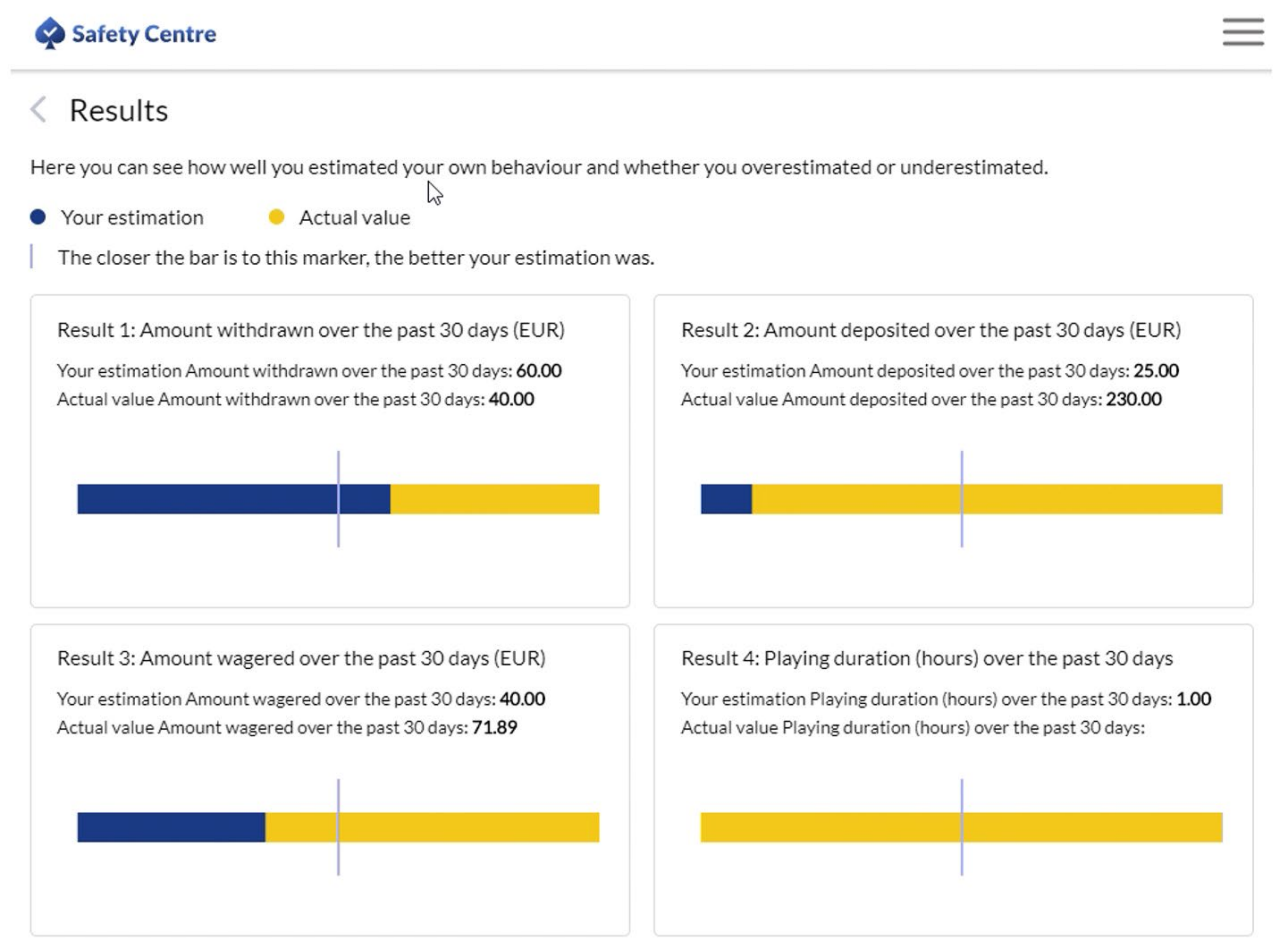
Feedback concerning the actual amount of money deposited compared to the estimated amount of money deposited on the online gambling website

Figures 1 and 2 show the self-reported amount of money deposited by a player and the feedback about the actual amount of money deposited by the gambling operator. In the example shown, the player estimated the amount of money deposited during the past 30 days was €25. After clicking the ‘Submit’ button, the player was then shown the actual amount of money deposited during the past 30 days which was €230. A horizontal bar displays the difference between the self-reported amount of many deposited and actual amount of money deposited. If both sections in the bar are equal sized, the self-reported amount of money deposited is the same as the actual amount of money deposited. In example shown, the section showing the self-reported amount of money deposited was much smaller because the player greatly underestimated the actual amount of money deposited.

Each record in the dataset contained the date gamblers estimated their deposit amount for the past 30 days. Only gamblers who deposited at least once during the past 30 days were included in the dataset. The dataset contained players’ gambling behavior from March 1, 2021 to February 28, 2023. In total there were 1,563 records, which means that some gamblers assessed their past 30-day amount of money deposited more than once. The dataset also included information about the gamblers’ age, gender, the aggregated gambling behavior in the 30 days prior to the self-assessment (actual amount of money deposited, actual amount of money withdrawn, and actual number of gambling days), and the gamblers’ estimations of their own monetary deposits within the past 30 days. Moreover, the actual amount of money deposited for the 30 days after the self-assessment was also included.

### Study Design

Because the dataset only included individuals who deposited money at least once during the past 30 days, the authors removed all gamblers from the dataset who reported that they never deposited any money within the past 30 days. The rationale behind this was to ensure that the dataset only contained gamblers who reported a reasonable estimate about their past monetary deposits. After this step, 693 records out of the 1,563 total records remained in the dataset (44%). The 693 records comprised 674 gamblers, which meant that a few gamblers assessed their amount of money deposited multiple times during the observation period. To measure the bias between the gamblers’ actual monetary deposits and their estimates, the following formula was used:

$$Relative\,\,bias = (estimated\,\,deposit - actual\,\,deposit)\,/\,actual{\rm{ }}deposit)*100$$

The relative bias measures the percentage deviation between actual and estimated monetary deposits. The minimum relative bias is -100% if players estimated their deposit to be zero. However, players who estimated their deposit to be zero were removed from the dataset. To exclude players with unreasonably high estimates, the authors excluded the top 5% of players with the highest estimated monetary deposit amount relative to their actual monetary deposits. This upper threshold was 1,704%, and players who overestimated their deposit amount by at least this amount were removed from the dataset. This left 658 self-assessments from 639 gamblers in the final dataset.

### Statistical Analysis

Several statistical tests were performed throughout the present study. To test whether a metric followed a normal distribution, the Shapiro-Wilk test was used. The tests reported a significant deviation from a normal distribution for the age (*W* = 097, *p* < .001), the amount of money deposited (*W* = 0.50, *p* < .001), the estimated amount of money deposited (*W* = 0.16, *p* < .001), the amount of money withdrawn (*W* = 0.27, *p* < .001), and the number of gambling active days (*W* = 0.86, *p* < .001) in the 30 days before the self-assessment. Finally, Bonferroni correction was used to account for multiple statistical tests per hypothesis.

#### Accuracy of Estimating the Total Amount Deposited (RQ1)

First, the authors calculated the percentage of gamblers who either underestimated, overestimated, or correctly estimated their monetary deposits. Second, for comparability with Heirene et al. (2022), the authors reported the same percentages, while allowing for a 10% deviation as an accurate estimate of the monetary deposits.

#### Association of Age and Gender with the Bias Types of the Estimates (RQ2)

To test whether there were gender or age differences between the types of gamblers who overestimated, correctly estimated, or underestimated their monetary deposits, various statistical tests were used. First, z-proportion tests were used to compare the proportions of accurate deposit estimations between males and females. Second, a Kruskal-Wallis test was used to assess overall differences in age between the estimation groups. Finally, pairwise Mann-Whitney U-tests were used to assess potential differences between pairs of groups.

#### Association Between the Gambling Behavior and the Bias of the Monetary Deposit Estimates (RQ3)

First, the authors applied a Kruskal-Wallis test to evaluate whether there were differences in the amount of money deposited in each group (i.e., gamblers who underestimated, correctly estimated, and overestimated their deposits). Second, to further investigate the relationship between the magnitude of the amount of money deposited and the type of bias, gamblers were categorized into ten equally sized groups according to their actual amount of money deposited in the 30 days prior to the self-assessment. Following this, the percentage of gamblers who underestimated, correctly estimated, and overestimated their monetary deposits was calculated for each group.

Third, two logistic regressions were fitted to evaluate which behavioral factors were associated with underestimating (first regression), and overestimating (second regression) the monetary deposits compared to gamblers who correctly estimated their deposits. Therefore, the first model was fitted on gamblers who underestimated or correctly estimated their monetary deposits, and the second model was fitted on gamblers who overestimated or correctly estimated their monetary deposits. The dependent variable indicated whether the gamblers underestimated (or overestimated) or correctly estimated their monetary deposits. The independent variables were the number of gambling days, the amount of money deposited, and the amount of money withdrawn in the 30 days prior to the self-assessment. Due to the skewed nature of the amount of money deposited and amount of money withdrawn, a log transformation was applied.

#### Impact of Feedback on the Estimation Bias on Subsequent Depositing (RQ4)

To evaluate the impact of feedback regarding the estimation bias on subsequent monetary depositing, paired Wilcoxon tests were used to compare the amount of money deposited 30 days before and after the self-assessment for each of the three bias types. Because three tests were performed, a Bonferroni-corrected alpha level of 0.016 (i.e., 0.05/3) was used to report statistical significance.

To evaluate whether the feedback about an overestimation or underestimation led to a change in the amount of money deposited within the next 30 days compared to gamblers who correctly estimated their deposits, two separate difference-in-differences regressions were used. Using this method, the actual intervention effects were separated from concurrent effects, which may have occurred even without the intervention. Furthermore, this method accounts for the differences between the gamblers who underestimate (or overestimate) their monetary deposits and the gamblers who correctly estimated their monetary deposits. The resulting regression model was as follows:

$$log\left(amount deposit\right) \sim {\beta }_{0}+{\beta }_{1}period+{\beta }_{2}intervention+{\beta }_{3}period:intervention$$

where the variable *period* describes whether a gambler’s monetary deposit was before or after the self-assessment, and the variable *intervention* describes whether a gambler underestimated or correctly estimated their monetary deposits. To evaluate whether overestimation had an effect, the same regression model was applied but, in this case, the variable *intervention* describes whether a gambler overestimated or correctly estimated their monetary deposits. In the above regression models, *β*_*1*_ estimates potential seasonal differences before and after the self-assessment, *β*_*2*_ estimates the differences in the 30 days before the self-assessment between gamblers who underestimated (or overestimated) their monetary deposits and gamblers who correctly estimated their monetary deposits, and *β*_*3*_ estimates the actual effect of the underestimation (or overestimation) on the gambler’s monetary deposits in the next 30 days after the self-assessment.

### Participants

The average age of the 639 gamblers was 40 years (SD = 11) and 287 were female (43%). Table 1 reports descriptive statistics for the gamblers estimated and actual amount of money deposited, as well as the relative bias. In self-assessment, 25% of the gamblers estimated their deposited amount to be less than €80. In reality, 25% of gamblers deposited at most €75. The estimated median amount was €230, and the actual median amount was €226. In self-assessment, 25% of gamblers estimated their amount deposited equal or larger than €600. In reality, 25% of gamblers deposited at least €560. The median relative bias was 0%. In 25% of the cases, the actual amount of money deposited was overestimated by at least 88%. In 25% of the cases, the actual amount of money deposited was underestimated by at least 37%.

**Table 1:** 25%, 50% and 75% percentile values for the estimated and actual amount of money deposited, the relative bias, the number of active gambling days, and the amount of money withdrawn

	Estimated amount of money deposited (€)	Actual amount of money deposited (€)	Relative bias	Active gambling days	Amount of money withdrawn (€)
Q1	80	75	-37%	2	0
Median	230	226	0%	5	0
Q3	600	560	88%	11	214
Maximum	70,000	13,088	1,700%	30	21,158

### Ethics

This study was performed in line with the principles of the Declaration of Helsinki and was approved by the last author’s university ethics committee.

## Results

### Accuracy of Monetary Deposit Estimations (RQ1)

Table 2 reports the percentage of gamblers who either underestimated, correctly estimated, or overestimated their monetary deposits. Out of the 658 self-assessments, 115 exactly matched the actual amount of money deposited (17.5%), 262 estimated their monetary deposits to be lower than the actual amount deposited (39.8%), and 281 estimated higher than the actual amount of money deposited (42.7%). Furthermore, Table 2 reports the same percentages using a 10% range in either direction to be counted as a correct estimation for comparability with Heirene et al. (2022). Using this range, one-quarter correctly estimated their deposits (25.7%), 35.9% underestimated, and 38.3% overestimated their monetary deposits.

**Table 2:** Percentage of gamblers who underestimated, correctly estimated, or overestimated their deposits. For comparison with Heirene et al. (2022), the same values are reported by allowing for a 10% deviation to be counted as a correct estimation

Percentage of gamblers	Underestimated amount of money deposited	Correctly estimated amount of money deposited	Overestimated amount of money deposited
Exact thresholds	39.8%	17.4%	42.7%
Allowing a 10% deviation	35.9%	25.7%	38.4%

### Gender and Age Differences in Monetary Deposit Estimations (RQ2)

Table 3 shows the proportion of male and female gamblers who either underestimated, correctly estimated, or overestimated their monetary deposits. Using the Bonferroni-corrected alpha level of 0.016 (i.e., 0.05/3), no significant differences were found between male and female gamblers in the prevalence of each type of estimation bias. More specifically, 37.1% of male and 43.4% of female players underestimated their monetary deposits (*z*=-1.63, *p* = .104), 16.4% of males and 18.9% of females provided correct estimates of their monetary deposits (*z*=-0.81, *p* = .419), and 46.4% of males and 37.7% of females overestimated their monetary deposits (*z* = 2.23, *p* = .026). There were differences in the group of players who overestimated their monetary deposits (*p* = .026). However, this result was not significant due to conservative adjustment by the Bonferroni correction for multiple testing.

**Table 3:** Percentage of male and female gamblers who underestimated, correctly estimated, or overestimated their monetary deposits. As three statistical tests were performed, significance is reported at the Bonferroni corrected alpha level of 0.05/3 = 0.016

	Underestimated amount of money deposited	Correctly estimated amount of money deposited	Overestimated amount of money deposited	Total
Males	37.1% (140)	16.4% (62)	46.4% (175)	100% (377)
Females	43.4% (122)	18.9% (53)	37.7% (106)	100% (281)
Z-test	*z*=-1.63, *p* = .104	*z*=-0.81; *p* = .419	*z* = 2.23, *p* = .026	

Using the Kruskal-Wallis test, there was a significant difference in the age of gamblers across the three types of estimation bias (*H* = 9.05, *p* = .011). To further investigate these differences, pairwise Mann-Whitney U-tests were conducted with Bonferroni-corrected alpha-levels of 0.016 (i.e., 0.05/3). These tests indicated a significant difference in age between gamblers who overestimated (*Median* = 38 years) and gamblers who underestimated (*Median* = 41 years) their monetary deposits (*U* = 41,998, *p* = .005). However, there were no significant differences between the ages of gamblers who correctly estimated (*Median* = 40 years) and underestimated (*U* = 15,159, *p* = .923), or gamblers who correctly estimated and overestimated their monetary deposits (*U* = 18,212, *p* = .047).

### Association Between Bias Type and Gambling Behavior (RQ3)

First, the differences in the amount of money deposited across the three types of estimation bias were examined. A Kruskal-Wallis test showed significant differences in the amount of money deposited between gamblers who underestimated, correctly estimated, and overestimated their deposits (*H* = 40.40, *p* < .001). Pairwise Mann-Whitney U-tests showed that the amount of money deposited was significantly higher for gamblers who underestimated their deposits (*Median*=€411) compared to those who overestimated their deposits (*Median*=€241; *U* = 28,865, *p* < .001). Similarly, the amount of money deposited was significantly higher for gamblers who overestimated their deposits compared to those who accurately estimated their deposits (*Median*=€120; *U* = 13,058, *p* = .001). These findings suggest that gamblers who accurately estimated their deposit amount deposited the least money, followed by those who overestimated their deposits, while those who underestimated their deposits had the highest monetary deposits.

**Fig. 3:**
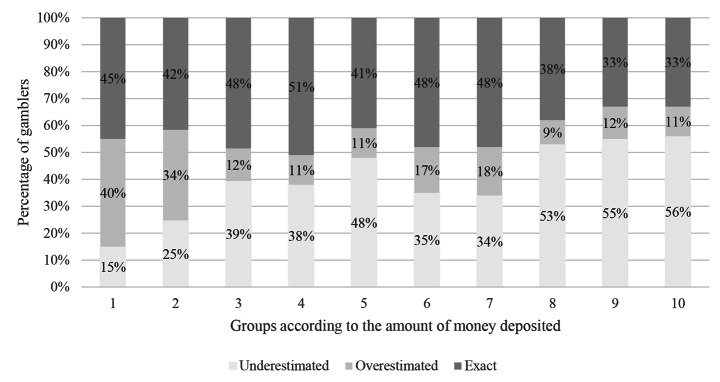
Percentage of gamblers who underestimated, exactly estimated, and overestimated their actual amount of money deposited for each of ten groups based on the actual amount of money deposited

To further investigate the relationship between the magnitude of the amount of money deposited and the tendency to underestimate, overestimate, or correctly estimate deposits, the players were categorized into ten equally sized groups according to the amount of money deposited in the 30 days prior to the self-assessment. Figure 3 displays the percentages of gamblers who underestimated, exactly estimated, and overestimated their actual amount of money deposited for each of the ten groups. The percentage of gamblers underestimating their losses increased from Group 1 to Group 10. More specifically, 15% of gamblers in Group 1 underestimated the amount of money deposited, and 56% of gamblers in Group 10 underestimated the amount of money deposited. On the other hand, 40% of gamblers in Group 1 overestimated the amount of money deposited, and 11% of gamblers in Group 10 overestimated their amount of money deposited. In Group 1, 45% of gamblers correctly estimated the amount of money deposited, which was the largest percentage across all groups. In Group 10, 33% of gamblers correctly estimated the amount of money deposited.

Two logistic regressions were performed to determine the association between the bias type and the gambling behavior. The first model evaluated the association between the gambling behavior and the tendency to underestimate monetary deposits. Table 4 reports the coefficients of the model. The log-transformed amount deposited, and the number of active gambling days were positive and significant. This means that a larger amount of money deposited, and a higher number of active gambling days were associated with a higher likelihood of underestimating monetary deposits. The coefficient for the amount of money withdrawn was not significant. The Cox and Snell (CS) pseudo *R*^*2*^ was 21.69%, which reflects adequate model fit.

**Table 4:** Logistic regression with the indicator variable whether the monetary deposits were underestimated or correctly estimated as dependent variable, and the gambling behavior (i.e., number of playing days, amount of money deposited, and amount of money withdrawn) in the 30 days prior to the self-assessment as independent variables

Variable	*β*	Std. error	*p*-value	CI
Intercept	-2.1955***	0.522	< 0.001	[-3.218, -1.173]
Number of playing days	0.1565***	0.031	< 0.001	[0.096, 0.217]
log(amount deposited)	0.3485***	0.098	< 0.001	[0.157, 0.540]
log(amount withdrawn)	0.0871	0.057	0.124	[-0.024, 0.198]
Cox and Snell *R*^2^	0.2169			

***p < .001, **p < .01, *p < .05

The second model evaluated the likelihood of overestimating monetary deposits. As shown in Table 5, the log-transformed amount of money withdrawn, and the number of active gambling days were positive and significant. This means that a larger amount of money withdrawn, and a higher number of active gambling days were associated with a higher likelihood of overestimating monetary deposits. The coefficient for the amount deposited was not significant. The CS *R*^*2*^ was 6.66%, which reflects a low model fit.

**Table 5:** Logistic regression with the indicator variable whether the monetary deposits were overestimated or correctly estimated as dependent variable, and the gambling behavior (i.e., number of playing days, amount of money deposited, and amount of money withdrawn) in the 30 days prior to the self-assessment as independent variables

Variable	*β*	Std. error	*p*-value	CI
Intercept	-0.2061	0.427	0.629	[-1.043, 0.631]
Number of playing days	0.0590*	0.027	0.029	[0.006, 0.112]
log(amount deposited)	0.1179	0.087	0.175	[-0.053, 0.288]
log(amount withdrawn)	0.1238*	0.053	0.020	[0.019, 0.228]
Cox and Snell *R*^2^	0.0666			

***p < .001, **p < .01, *p < .05

### Effect of Feedback on Monetary Depositing Behavior (RQ4)

A paired Wilcoxon test was used to examine the differences in monetary deposit amounts 30 days before and after the self-assessment across the three types of estimation bias. For gamblers who overestimated their monetary deposits, the median deposit amount significantly decreased from €241 before the self-assessment to €82 afterwards (*W* = 27,532, *p* < .001). For gamblers who correctly estimated their deposits, the median deposit amount also significantly decreased from €120 to €35 (*W* = 4,174, *p* = .006). Lastly, gamblers who underestimated their monetary deposits also experienced a significant reduction in the median deposit amount from €411 to €111 (*W* = 22,775, *p* < .001). These results suggest a notable reduction in amount of money deposited following the self-assessment for all gamblers.

To evaluate the impact of providing feedback on whether gamblers overestimated or underestimated their monetary deposits, two separate difference-in-differences regressions were performed to examine changes in the amount of money deposited within the next 30 days after the self-assessment. The first model assessed whether underestimation had an effect on future monetary deposits, and the second model assessed whether overestimation had an effect on future monetary deposits compared to the gamblers who correctly estimated their monetary deposits. Tables 6 and 7 show the corresponding coefficients of the regressions. First, both coefficients for the *intervention* variable were positive and significant, which means that gamblers who overestimated and underestimated their monetary deposits, deposited more money in the 30 days prior to the self-assessment than gamblers who correctly estimated their deposits. These findings confirm the results of RQ3 and corroborate the relationship between estimation bias and the amount of money deposited by gamblers.

**Table 6:** Results of the difference-in-differences regression to measure the effect of underestimation on the amount of money deposited in the next 30 days after the self-assessment

Variable	*β*	Std. error	*p*-value	CI
Intercept	4.858***	0.210	< 0.001	[4.445, 5.270]
Intervention	1.046***	0.252	< 0.000	[0.551, 1.541]
Period	-1.792***	0.297	< 0.001	[-2.376, -1.209]
Intervention:period	-0.061	0.357	0.863	[-0.761, 0.639]
*R* ^2^	0.173			

***p < .001, **p < .01, *p < .05

**Table 7:** Results of the difference-in-differences regression to measure the effect of overestimation on the amount of money deposited in the next 30 days after the self-assessment

Variable	*β*	Std. error	*p*-value	CI
Intercept	4.858***	0.208	< 0.001	[4.449, 5.266]
Intervention	0.535*	0.247	0.031	[0.050, 1.020]
Period	-1.792***	0.294	< 0.001	[-2.370, -1.214]
Intervention:period	-0.111	0.349	0.751	[-0.797, 0.575]
*R* ^2^	0.157			

***p < .001, **p < .01, *p < .05

The coefficients for the *period* variable were both negative and significant, which indicates that all gamblers (i.e., those who overestimated, underestimated, and correctly estimated their monetary deposits) significantly decreased the amount of money they deposited after the self-assessment. This finding is in line with the results of the paired Wilcoxon tests, which also reported a significant reduction across all three types of estimation biases. Finally, both the coefficients for the interaction between *period* and *intervention* were non-significant, which suggest that gamblers who underestimated or overestimated their monetary deposits did not decrease their deposits more (or less) than gamblers who correctly estimated their deposits. This means that the feedback concerning overestimation or underestimation did not significantly change the amount of money deposited compared to gamblers who correctly estimated their amount of money deposited when taking into account the general trend of the reduction in the amount of money deposited after the self-assessment.

## Discussion

The goal of the present study was to determine whether online gamblers were able to accurately estimate their amount of money deposited for the past 30 days, what factors were associated with a specific type of bias, and whether feedback about the bias of the estimate had an impact on subsequent monetary depositing. Three previous studies have compared the self-reported loss and the actual losses among real-world online gamblers (i.e., Auer & Griffiths, 2017; Braverman et al., 2014; Heirene et al., 2022). However, the present study is the first study to compare self-reported amount of money deposited to the actual amount of money deposited using a real-world sample of online gamblers.

### Can Gamblers Accurately Estimate Their Total Amount Deposited in the Last 30 days? (RQ1)

The estimated median amount of money deposited in the present study was €230 and the actual median amount of money deposited was €226. Out of the 658 self-assessments, 115 correctly estimated the actual amount of money deposited (17.5%), 262 estimated lower than the actual amount of money deposited (39.8%), and 281 estimated higher than the actual amount of money deposited (42.7%). Braverman et al. (2014) found that only 13% and 7% of participants accurately recalled their past three-month and past 12-month outcomes, respectively. Also, Heirene et al. (2022) reported that only 4.09% of participants assessed their losses accurately within a 10% margin of their actual outcome. Only two participants were completely accurate (0.39%). Heirene et al. (2022) also reported that 64.79% of participants were most likely to underestimate their losses, whereas Braverman et al. (2014) reported percentages between 34% and 40%. The relatively large percentage of gamblers who accurately estimated their amount deposited in the present study could be related to the different estimation metric. The amount of money lost estimated in the previous studies was based on each bet and win, which means that there was usually a much larger number of transactions to consider compared to the number of deposits. In a study of pop-up messages, Auer and Griffiths (2017) reported that gamblers can gamble approximately 1,000 times during one hour of consecutive play on online slots. Therefore, the estimation for the amount of money lost in the past 30 days is consequently based on a very large number of gambling events. In contrast, another study by Auer and Griffiths (2022) found that gamblers on average deposit money only once per session, but place multiple bets in a session, which highlights that the estimation of the amount of money deposited is based on a much smaller number of events than the estimation of the amount of money lost. Moreover, gamblers typically deposit round amounts like €10, €20, €100, etc., whereas the amount of money lost can be any decimal number. Therefore, it might also be easier to guess these natural numbers than a decimal number.

### Are Age and Gender Associated with the Accuracy of Deposit Amount Estimation? (RQ2)

The results of the present study showed no significant differences between male and female gamblers concerning underestimation and accurate estimation of monetary deposits. However, a potential difference emerged for gamblers who overestimated their monetary deposits, although it did not reach statistical significance after Bonferroni correction for multiple testing. Overall, these findings are in line with Heirene et al. (2022) who also reported no significant differences between genders in the percentage discrepancy of self-reported and actual net outcome.

In terms of age, a significant difference was found between gamblers who overestimated and those who underestimated their monetary deposits, with gamblers who overestimated their deposits being younger. However, no significant differences were observed in age when comparing gamblers who correctly estimated their monetary deposits to those who either overestimated or underestimated their deposits. One explanation for the age differences could be that younger individuals may be more cautious about their spending due to financial constraints, leading them to perceive their monetary deposits as higher than they were in reality. Further research is needed to investigate the role of age and other demographic variables with regards to estimation biases.

### Does the Gambling Behavior Impact the Accuracy of Deposit Amount Estimation? (RQ3)

The results suggested that gamblers who accurately estimated their deposits deposited the least amount of money, followed by those who overestimated their deposits, while those who underestimated their deposits had the highest amount of money deposited. Moreover, the logistic regression model indicated that there was a positive correlation between the amount of money deposited and the number of active days with the likelihood to underestimate the deposits. In other words, the more players deposited and the more active gambling days they had, the higher the probability that they underestimated the amount of money deposited. This is in line with both the findings of Auer and Griffiths (2017) and Braverman et al. (2014) who also found that the estimation bias increased with gambling intensity. Heirene et al. (2022) found that among net losers, those who lost more money were more likely to underestimate their losses. Moreover, higher actual betting frequencies were associated with underestimating betting. These results are in line with the findings of the present study.

### Does Feedback Whether Gamblers Over- or Underestimate Their Deposits Impact Subsequent Depositing? (RQ4)

The present study is the first to also analyze the effect of feedback about the estimation bias on subsequent depositing behavior. A difference-in-differences regression model was applied to consider the general trend of depositing money before and after the self-assessment. It showed that gamblers deposited significantly less money after the self-assessment, irrespective of how accurately they estimated their monetary deposits, which was also confirmed by the results of the paired Wilcoxon tests. Considering the overall reduction in deposits, there was no additional effect of the self-assessment and the corresponding feedback about the estimation bias. In other words, the feedback as to whether gamblers overestimated or underestimated their deposits did not lead to any additional significant changes in the amount of money deposited when considering the overall reduction in deposits after the self-assessment.

One explanation could be that gamblers who voluntarily took the self-assessment were more aware of their gambling and therefore made the decision to control their gambling by exploring the operator’s responsible gambling section where they completed the self-assessment. Future work could conduct controlled experiments in which participants are randomly assigned to receive feedback on either their estimation bias or a control condition, which would allow for a more robust assessment of the effect of feedback on subsequent monetary depositing behavior independent of the self-selection bias.

Another explanation for the lack of an additional effect of feedback concerning the estimation bias could be that gamblers were required to estimate several aspects of their activity, including amounts withdrawn, amounts wagered, and how long they had gambled. Therefore, feedback regarding a potential bias in their estimates might have affected other behaviors. For example, gamblers might have adjusted their playing duration in response to the feedback, an aspect not analyzed in the current study.

Considering these findings, future research might benefit from analyzing the impact of such feedback on other behavioral variables, as well as providing an improved visual representation of feedback that is more intuitive and less overwhelming. Presenting feedback in a user-friendly, easily digestible manner may increase the effectiveness of interventions designed to reduce gambling behavior. Given that gamblers interact with a wealth of information, it is critical that the feedback they receive is clear, engaging, unambiguous, and contextually relevant. This is an important avenue for future research and has the potential to increase the efficacy of feedback as a tool for promoting responsible gambling behavior.

### Limitations

The present study has a number of limitations. First, a different metric was used for the self-assessment, which makes it difficult to directly compare the results here to other studies, which used the amount of money lost instead of the amount of money deposited. However, the amount of money deposited may be a more suitable metric for gamblers to estimate their gambling expenses given the lower event frequency (i.e., the number of deposits is much lower than the number of gambles/bets). Second, the present results suggested that gamblers deposited significantly less money after the self-assessment, irrespective of how accurately they estimated their monetary deposits. This effect may be explained by a self-selection bias since players voluntarily chose to do the self-assessment. Third, all the data were from a relatively small number of gamblers from just one gambling operator’s website. Therefore, replication with larger numbers of gamblers across different operator’s websites is needed.

## Conclusions

On average, as demonstrated by the median values, players were able to estimate fairly accurately how much money they had deposited in the past 30 days. However, the higher the amount of money deposited, the more likely gamblers underestimated the actual amount of money deposited. This is worrisome given that the extent of the discrepancy between self-reported and actual outcomes is positively associated with problem gambling (Braverman et al., 2014). For players to avoid overspending, they need to be able to keep track of their expenditure. For this reason, it is important that gamblers have easy access to information regarding their expenditure when they gamble. Information is an integral part of the Reno model which is a framework for safer gambling for policymakers, regulators, and operators (Laudouceur et al., 2016).

Based on these findings, future research should conduct controlled experiments to robustly assess the effect of feedback on subsequent behavior. In addition, given that feedback might also affect other gambling behaviors, such as playing duration, examining these additional behavioral variables could also provide valuable insights. Improving the visual presentation of feedback to be more intuitive might also increase its efficacy as a responsible gambling intervention. Given that in recent years many jurisdictions (e.g., UK, Germany, Sweden, Denmark, Spain) have made it mandatory for gambling operators to give players feedback about their actual gambling behavior, it is recommended that other large gambling markets, such as the US, follow such examples.

## Data Availability

Not applicable.
